# A simple new approach for mapping an ultrasonic tank for sonochemistry

**DOI:** 10.1016/j.ultsonch.2024.106940

**Published:** 2024-05-31

**Authors:** Timothy J. Mason, Daniela Ghimpeteanu, Ioan Călinescu, Mircea Vinatoru, Adrian Trifan

**Affiliations:** a College of Engineering, Environment and Science, Coventry University, Coventry, United Kingdom; b Bioresource Department, Politehnica Bucuresti National University of Science and Technology, RO-060042, Bucharest, Romania

**Keywords:** Mapping, Dosimetry, Ultrasonic Capillary Effect, Calorimetry, Iodimetry, Cavitometer, Aluminium foil perforation

## Abstract

The most used piece of equipment for sonochemistry is the ultrasonic cleaning bath. However, what is sometimes forgotten by scientists new to sonochemistry is the vital importance of the shape and positioning of any reaction vessel in the bath to obtain the most efficient and reproducible results. In experiments using an ultrasonic bath, a glass vessel (reactor) is inserted into the water contained in the bath. The water acts as the coupling medium for the transfer of acoustic energy from the transducer to the vessel (termed indirect sonication). The position of the reaction vessel above the base of the US bath can change the energy transmitted into it over a wide range of values (in our system between 100–500 J). We have carried out a study of the vertical distribution of the ultrasound field in a common type of ultrasound bath, comparing conventional sonochemistry dosimeters with a new and very simple approach using the Ultrasonic Capillary Effect (UCE) which can be performed in any laboratory. The technique involves the use of a capillary tube, to locate the vertical positions of acoustic pressure maxima above a single transducer on the base of the bath. The results are compared with those obtained using calorimetry, iodimetry, a cavitometer and the perforation of aluminium foil. The results show that the optimum position for the reaction vessel can be located very simply using UCE.

## Graphical abstract

**Figure:**
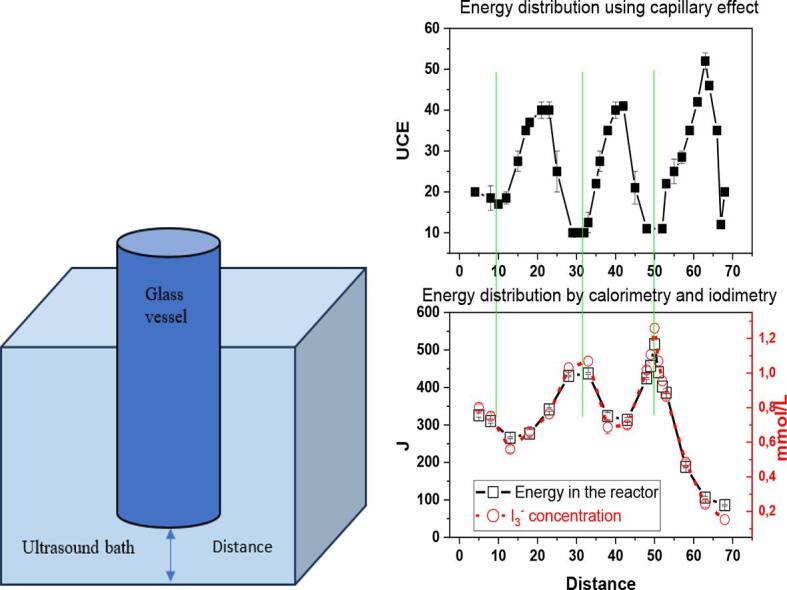

## 1 Introduction

Sonochemistry, as a defined subject, has a surprisingly short history of about 50 years and the first meeting entirely devoted to it took place in 1986 [1]. Its origins, however, date back to the early 20th century. In 1927 Alfred Loomis reported on two aspects of the effects of high frequency sound, one with Robert Wood, involving the physical and biological effects [2] and the second, with William Richards, on the chemical effects [3]. Some 20 years later Alfred Weissler, a pioneer in the study of uses of ultrasound in chemistry, published a number of papers in this field amongst which was a seminal review of the subject in the Journal of Chemical Education in 1948 [4].

One of the early concerns in sonochemistry at a practical laboratory level was the problem of reproducing experiments performed using different ultrasonic equipment. In a paper published in 1996 Kimura et. al., pointed out that: “It is often difficult to compare the sonochemical results reported from different laboratories. This difficulty is well known as the reproducibility problem in sonochemistry” [5].

From the beginning of studies in sonochemistry research, the simplest and most widely used source of ultrasound has been the ultrasonic cleaning bath. There are two major considerations when using such a bath to optimize the effects of ultrasound and these are to find (a) the best position for the reaction vessel in the tank and (b) to find the best shape of reaction vessel to allow the maximum transfer of acoustic energy from the bath water into the vessel [6]:

(a) A simple method for determining the best position for reaction vessel location is to find where the maximum disturbance is observed on the surface of the reaction mixture in terms of its horizontal and vertical positions in the bath. Once the correct position has been identified, it is a simple matter to arrange stands and clamps in order to locate it in the same location. Essentially, this will act as a template for future reactions since repeated use of the same vessel in the same location in the bath should give reasonably reproducible results.
(b) A method for the determination of the best glassware design involves the use of a sonochemical dosimeter e.g. the oxidation of potassium iodide. Using the same reaction volume of 50 cm^3^ in differently shaped vessels it was determined that a vessel with a flat glass base at right angles to the propagating sound wave was better than a vessel with rounded base [7].

There are many articles that refer to the mapping of ultrasound baths, they usually refer to baths with several transducers and the methods can involve a cavitometer [8], iodometry [9], the efficiency with which natural compounds are extracted [9], [10] or a hydrophone [11]. The possibility of predicting the cavitational activity by solving the wave equation in two different geometries of sonochemical reactors was also suggested [12]. In this current work, our aim is to extend these studies to determine the energy distribution in an ultrasonic bath at different depths using a very simple methodology based on the ultrasonic capillary effect. This new method is available in all chemical laboratories and can be used to determine the positions for maximal sonochemical activity. The method is compared to existing dosimetries: calorimetry, the oxidation of iodide and results using a commercially available cavitometer.

The UCE method of mapping an ultrasonic tank can be used by scientists in any laboratory whatever their level of expertise in sonochemistry.

## 2 Materials and methods

**Fig. 1:**
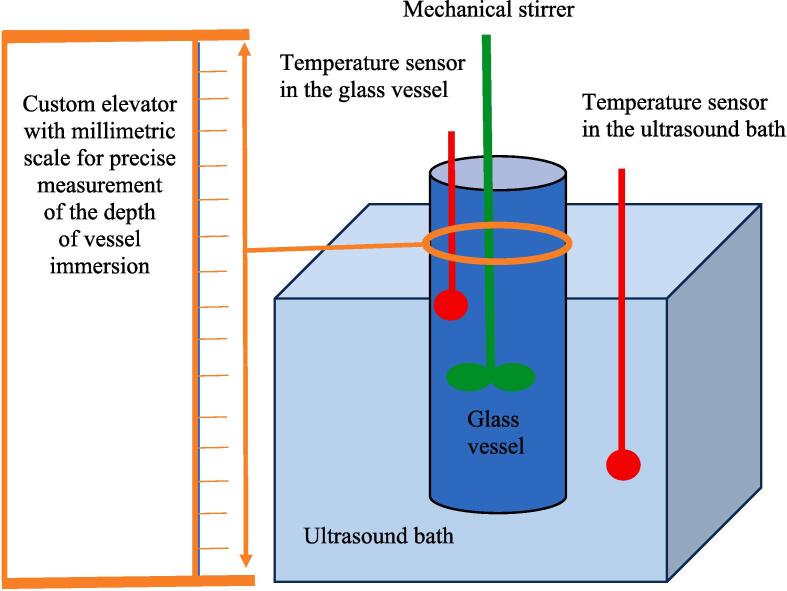
Experimental set-up used for dosimetry of an ultrasonic bath on a vertical axis using a glass reactor.

**Fig. 2:**
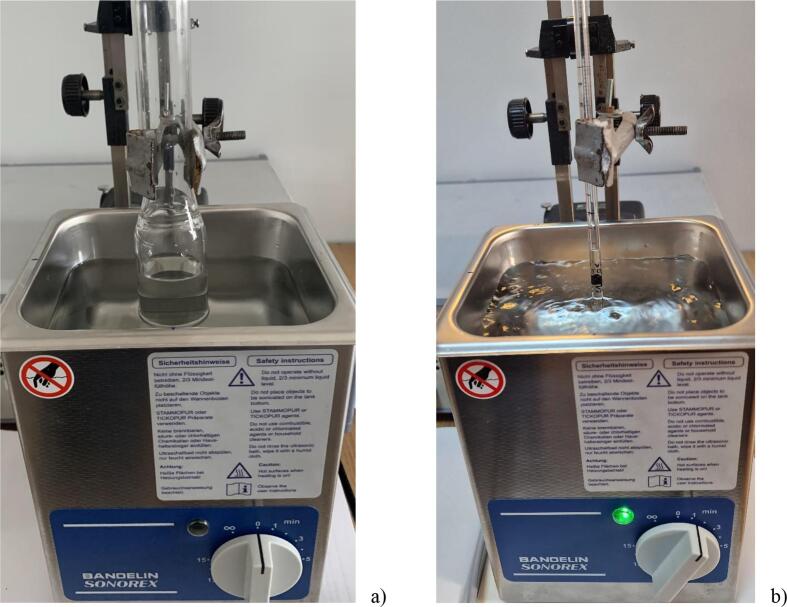
Images of the experimental set-up: a) the Berzelius vessel in the US bath, b) the capillary tube in the US bath.

A Bandelin Sonorex RK ultrasonic bath with single transducer (maximum power 80 W, operational frequency 37 kHz, transducer diameter 49.8 mm) filled with 1.2 dm^3^ tap water, was used for all experiments. A custom-made vessel support (Fig. 1), with a mechanical system able to move the reaction vessel or capillary tube in vertical plane, was used to achieve precise immersion depths for the glass reactor in the bath. The initial height of the liquid layer in the bath was 68 mm and after full immersion of the glass reactor (5 mm from the base) the height was raised by only 2.8 mm. The reactor was a flat bottom glass cylinder (50 cm^3^ nominal volume) shaped like a standard Berzelius vessel. For the experiments 35 cm^3^ of liquid (water/potassium iodide solution) were added to give a height of liquid inside the reactor vessel of 43 mm. The vessel has an external diameter of 35 mm and the thickness of the glass walls is 2 mm (Fig. 2a).

The capillary tube (Fig. 2b) was a standard graduated 2 cm^3^ pipette (Labbox MPIA-002–005) with a length of 320 mm, an inner diameter of 3.2 mm and a glass wall thickness of 1.8 mm. The pipette was positioned vertically with the tip out of the water. It is preferable to use a capillary tube with a diameter of about 3 mm because with such a tube, the capillarity effect in the absence of ultrasounds (US) is low and the water level changes rapidly when the US is switched on or off.

**Fig. 3:**
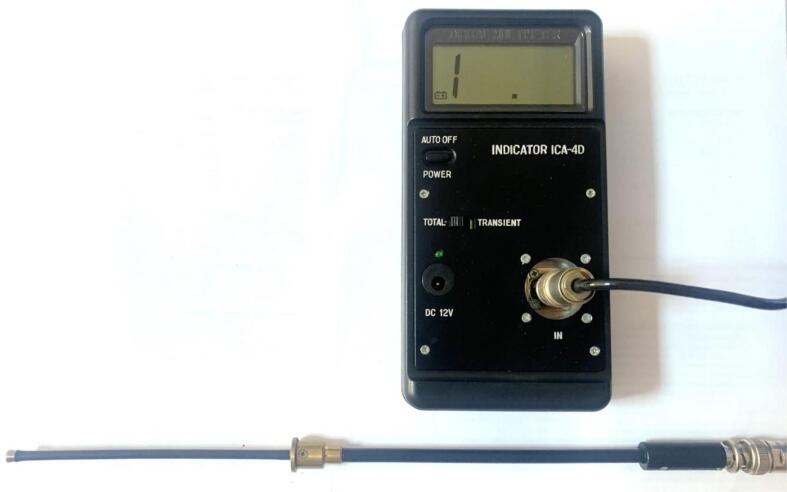
Cavitometer ICA −4D.

The cavitometer used was an ICA-4D (Fig. 3) which was developed and manufactured by the Belarusian State University of Informatics and Radioelectronics. (BSUIR, Minsk, Belarus). The probe is a 51 cm long stainless-steel tube with an elastic ethylene propylene diene monomer (EPDM) half sphere on one end. The impact of cavitation jets hitting the surface of the protecting half sphere is conducted to the piezo sensor mounted behind it and the resulting signal is monitored.

The foil test was carried out using a wire support (1 mm diameter) to fix and hold in place the aluminum foil (80x50 mm).

For calorimetric measurements, two temperature sensors connected to a high precision interface (IPC DAS, ET-7018Z) with 0.01 degrees accuracy (±0.1 % of FSR or better) were used to monitor the temperatures in the glass reactor (filled with 35 cm^3^ of water). The measurements were made only after the temperatures in the bath and reaction vessel had equilibrated to the same value. During the measurements, the thermocouples were removed and the ultrasound started for a period of 30 s. The temperature was then measured with the ultrasound switched off and the thermocouples in place. No stirrers were used in the reactor or tank during sonication because bulk movement of the liquid can disturb the acoustic field by scattering of ultrasonic waves [11]. All measurements were performed by placing either the capillary, the cavitometer, the aluminum foil or the reaction vessel in the centre of the bath directly above the transducer. All adjustments to position were on the vertical axis, at 5 mm increments (see Fig. 1, Fig. 2).

Measurements at each depth were performed in triplicate and the data are presented as average values together with variation limits. Equation (1) is used for the calorimetric determinations of the energy input into liquid contained in a vessel that is dipped into the ultrasonic bath:

(1) E=m∗Cp∗ΔT

where:

E = energy in the ultrasonic bath or glass vessel (J).

*m* = mass (kg).

*Cp* = specific heat capacity of the used liquid (J*kg^−1^*K^−1^).

*ΔT* = temperature difference (K).

Potassium iodide (analytical grade) was obtained from Merck and used as received. Iodine dosimetry was performed using the same volume of liquid in the same cylindrical glass reactor as used in the calorimetry measurements (Fig. 1). A volume of 35 cm^3^ potassium iodide solution (0.1 mol/dm^3^) was loaded into the reactor vessel and immersed at the same series of depths in the ultrasonic tank as used in calorimetry. The determination of I_3_^‾^ concentration was carried out by measuring the absorbance at 355 nm using a UV–VIS spectrophotometer (Shimadzu UVmini-1240) [4]. The concentrations of I_3_^‾^ were determined using equation (2) [13].

(2) $$[I_{3}^{-}]=abs/\varepsilon$$

where:

[ I_3_^‾^ ] = concentration of I_3_^‾^ ions (mol/dm^3^).

*abs* = the absorbance at 355 nm.

*ε* = 26.2 (dm^3^/mol*cm) [13].

## 3 Results and discussions

The methods used to determine the acoustic activity in terms of depth in the ultrasonic bath can be divided into two types, those which are based on the physical effects of cavitation (capillary rise, cavitometer and the perforation of foil) and those which are commonly used as dosimeters in sonochemistry (calorimetry and the liberation of iodine). The physical effects of cavitation were determined in the US bath containing only water, and without the glass reactor. The dosimetry determinations were made in liquids contained in the Berzelius glass vessel. (Fig. 2a).

### 3.1 Methods which use the physical effects of cavitation

#### 3.1.1 The use of capillary rise for monitoring acoustic pressure

The observation that water will rise within a capillary tube when it is dipped below the surface is over 300 years old [14]. Some 250 years later Nikolai Dezhkunov reported that ultrasound increased the capillary rise which he termed the ultrasonic capillary effect (UCE) [15]. He suggested that the most likely mechanism for UCE involved cavitation because the thresholds for both the initiation of cavitation and that of ultrasonically induced capillary rise virtually coincided. He also found that there was a quantitative agreement between UCE and the multibubble sonoluminescence emissions with increased amplitude of vibration [16]. UCE should not be confused with U-effect: the electrical potential developed between the ends of a capillary containing an electrolyte when ultrasonic vibrations are passed through [17].

**Fig. 4:**
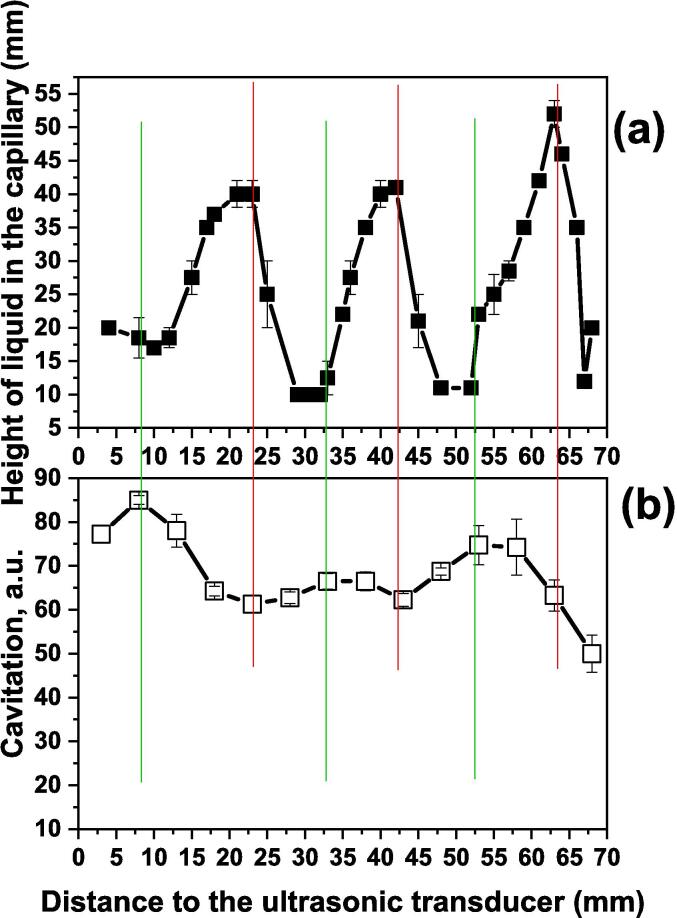
**(a)** The height of the liquid rise in the capillary and (b) the cavitometer signal at different distances to the transducer.

We report here another property of UCE which relates to the correlation between the height of UCE rise in the capillary to the depth of immersion of the capillary in an ultrasonic bath. The influence of distance between the transducer and the tip of the immersed capillary tube on the height of the liquid in the tube is shown in Fig. 4a. The depth of immersion was in small steps (each of only a few mm) to better establish the trend in capillary rise. The maxima in column height of liquid in the tube were at distances of 22, 42 and 62 mm. The wavelength (λ) of sound in water at an ultrasonic frequency of 35 kHz is 40 mm, and therefore the distance between antinodes (or nodes) is 20 mm (i.e. λ/2). This clearly indicates that the capillary tube experimental maxima correspond with either antinodal or nodal points within the tank. These observations suggested to us the possibility of using ultrasonic capillary rise as an indicator of the amount of cavitational activity at different depths in the bath.

#### 3.1.2 Cavitometer measurements

Using the same arrangement as for the capillary rise experiments (Fig. 2b), the results for the cavitometer are shown in Fig. 4b. As in the case of capillary measurements the cavitometer results also show three maxima. However, there is a remarkable difference between them because the maximum values recorded from the UFE correspond with the minimum from the cavitometer and the minima of UFE correspond with the maxima at the cavitometer. This apparent anomaly can be explained in terms of the standing wave set up in the bath water and the difference between the two methods of measurement. A cavitometer records zones of cavitation activity which is similar to the observation of sonoluminescence bands in a standing wave pattern. The latter study concluded that cavitation activity, the cause of sonoluminescence, correlated closely with the pressure antinodes in the standing wave pattern [18].

It is interesting to note that the positions of maximum cavitometer readings also correspond to those obtained for maxima in heat generation and free radical formation (see Section 3.2). As a result, this implies that the peaks observed in capillary rise are driven by acoustic pressure maxima at the standing wave nodes. This also suggests that the peaks observed in capillary rise are driven by acoustic pressure maxima at the standing wave nodes.

#### 3.1.3 Aluminum foil test

A commonly used assessment of the distribution of the cavitation intensity in an ultrasonic bath is the aluminum foil test [19], [20], [21]. The method is not quantitative, but it provides indications of the active areas within the bath. In our hands, the damage to the foil (Fig. 7b) shows multiple areas of erosion located at different distances from the ultrasound transducer: 8–14; 23–25; 41–43 and 57–59 mm. The most affected area is that located closest to the transducer. There is a general similarity between the distances between areas of foil damage and the pattern of activity obtained with capillary and cavitometer measurements, but they are not an exact match. We do not have a definitive explanation for this, but it may be due to the disruption of the transmission of ultrasound in the bath by the presence of the aluminum foil and its support system. The foil registers all positions of cavitation damage over its complete immersed length at the same time. In contrast the cavitometer, capillary and dosimeters record individual maxima at different distances with, in each case, clear unobstructed water between the transducer and the detection system. See (Fig. 6).

### 3.2 Dosimetry methods used for sonochemistry

**Fig. 5:**
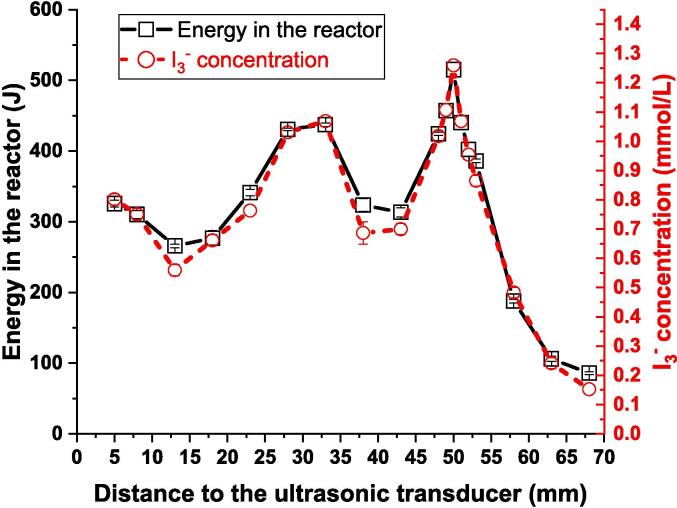
Correlation between calorimetry and iodimetry in the standard flat bottom Berzelius vessel, as a function of the distance from the ultrasonic transducer.

The two most used dosimeters in sonochemistry are calorimetry and the oxidation of iodide to iodine. Both have a connection to the amount of acoustic cavitation produced by sonication, but the former is the result of physical effects while the latter is chemical. We report a comparison of these dosimeters using the same volume of liquid, the same glass reactor and the same US exposure time of 30 s. As shown by the data presented graphically in Fig. 5, there is a remarkable similarity between the results of the two sets of experiments. In both cases, the maximum effect occurs near the surface, at 50 mm from the base with another, smaller, maximum at 30 mm. Iodometry has been used, in the past, for the mapping of acoustic fields [11]. However, we believe that this is the first published example of its correlation with calorimetry.

**Fig. 6:**
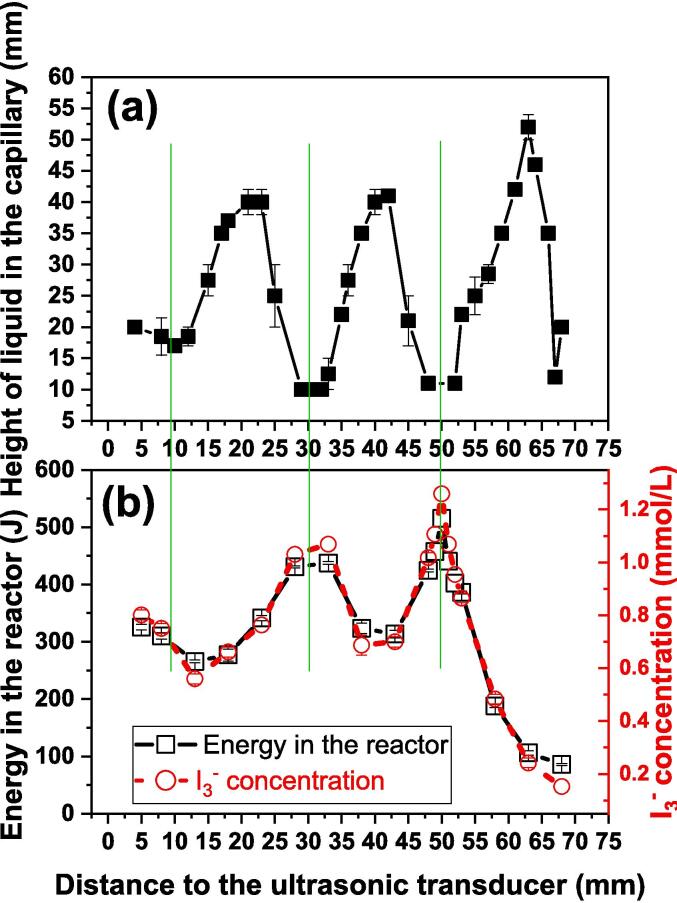
Comparison of capillary measurement (a) and sonochemistry dosimeters (b).

### 3.3 Comparison of methods used to determine acoustic activity

There is a correlation between the maximum values shown in the cavitometer measurements (Fig. 4) and the maxima for both the calorimetry and iodimetry (Fig. 5). This is to be expected because the dosimeters require maximum transfer of cavitational energy through the base of the reaction vessel. The results from the UCE measurements show that these positions also correspond to minima in capillary rise i.e. lowest acoustic pressures. These minima on the UCE measurements are clearly defined and very easily recorded and so constitute a valuable new method for sonochemistry mapping of active cavitation zones.

**Fig. 7:**
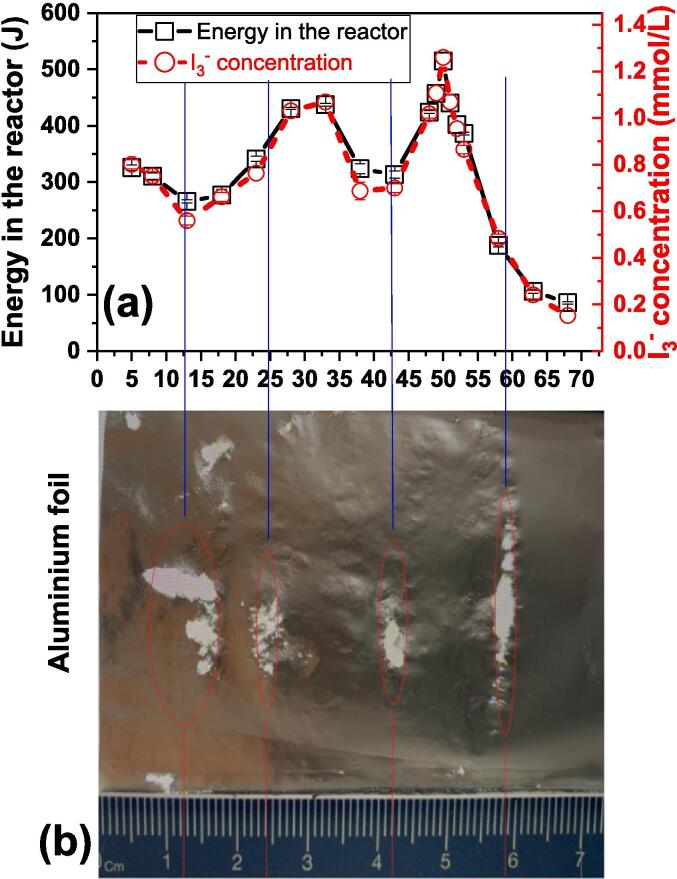
Comparison of sonochemistry dosimeters (a) and foil perforation (b).

A comparison between foil perforation and sonochemistry dosimeters is less clear (Fig. 7). There appears to be no absolute correlation. The possible reasons for this have been outlined in section 3.1.3 above and support for this comes from the observation that the character of the erosion zones was found to be somewhat dependent upon the width of foil used in the test.

## 4 Conclusions

For the sonochemist, the mapping of an ultrasonic tank is important from the point of view of understanding the acoustic cavitation distribution. The use of dosimeters based on iodine or calorimetry are accepted and used widely. A minor problem with both is that they are time-consuming. A cavitometer represents an alternative and rapid methodology but requires the purchase of a dedicated instrument – which can be expensive. The capillary method is both rapid and inexpensive, providing a simple way of mapping for any laboratory. We believe that future developments of this method could be adopted widely by sonochemists.

## Funding

This work was supported by Competitiveness Operational Programme 2014–2020, Action 1.1.4: Attracting high-level personnel from abroad in order to enhance the RD capacity, project: P_37_471, „Ultrasonic/Microwave Nonconventional Techniques as new tools for nonchemical and chemical processes”, financed by contract: 47/05.09.2016.

## CRediT authorship contribution statement

**Timothy J. Mason:** Supervision, Conceptualization. **Daniela Ghimpeteanu:** Investigation. **Ioan Călinescu:** Writing – original draft, Data curation, Conceptualization. **Mircea Vinatoru:** Validation, Methodology. **Adrian Trifan:** Writing – original draft, Visualization, Investigation, Formal analysis, Data curation.

## Declaration of competing interest

The authors declare that they have no known competing financial interests or personal relationships that could have appeared to influence the work reported in this paper.
